# Molecularly Imprinted Polymer Materials as Selective Recognition Sorbents for Explosives: A Review

**DOI:** 10.3390/polym11050888

**Published:** 2019-05-15

**Authors:** Mashaalah Zarejousheghani, Wilhelm Lorenz, Paula Vanninen, Taher Alizadeh, Malcolm Cämmerer, Helko Borsdorf

**Affiliations:** 1 UFZ-Helmholtz Centre for Environmental Research, Department Monitoring and Exploration Technologies, Permoserstraße 15, D-04318 Leipzig, Germany; malcolm.caemmerer@ufz.de (M.C.); helko.borsdorf@ufz.de (H.B.); 2 Institute of Chemistry, Food Chemistry and Environmental Chemistry, Martin-Luther-University Halle-Wittenberg, D-06120 Halle, Germany; wilhelm.lorenz@chemie.uni-halle.de; 3 VERIFIN, Finnish Institute for Verification of The Chemical Weapons Convention, Department of Chemistry, University of Helsinki, FI-00014 Helsinki Finland; paula.vanninen@helsinki.fi; 4 Department of Analytical Chemistry, Faculty of Chemistry, University College of Science, University of Tehran, 1417466191 Tehran, Iran; talizadeh@ut.ac.ir

**Keywords:** molecularly imprinted polymer, explosive, explosive marker, nitroaromatic compounds, trinitrotoluene, TNT, dinitrotoluene, trinitrophenol, sensors, sample preparation

## Abstract

Explosives are of significant interest to homeland security departments and forensic investigations. Fast, sensitive and selective detection of these chemicals is of great concern for security purposes as well as for triage and decontamination in contaminated areas. To this end, selective sorbents with fast binding kinetics and high binding capacity, either in combination with a sensor transducer or a sampling/sample-preparation method, are required. Molecularly imprinted polymers (MIPs) show promise as cost-effective and rugged artificial selective sorbents, which have a wide variety of applications. This manuscript reviews the innovative strategies developed in 57 manuscripts (published from 2006 to 2019) to use MIP materials for explosives. To the best of our knowledge, there are currently no commercially available MIP-modified sensors or sample preparation methods for explosives in the market. We believe that this review provides information to give insight into the future prospects and potential commercialization of such materials. We warn the readers of the hazards of working with explosives.

## 1. Introduction

Molecularly imprinted polymer (MIP) materials are highly cross-linked organic/inorganic co-polymers in which cavities (recognition sites) are imprinted for template molecules, whether explosive molecules or their nonexplosive structural-analogs, as dummy templates. The fundamental principles of MIP-technology can be found in other reviews [1,2,3,4,5]. The advantageous qualities of MIP materials (e.g., low cost, ease of engineering, simplicity of production, potential reusability, physical/chemical stability and their applicability for a wide range of targets) make their applications remarkably widespread [6,7,8,9,10,11,12]. MIPs have been used for the selective enrichment of explosives and explosive markers and, as shown in Table 1, nitroaromatic compounds (NACs) are the most important class of chemicals used in these studies. The presence of the nitro groups in NACs cause the aromatic ring to have an electron-deficient character. This means NACs can interact with electron-rich monomers via π–π interaction and charge-transfer interaction. The methyl group of trinitrotoluene (TNT) can even be deprotonated e.g., by basic amines in the Brønsted-Lowry acid-base reaction.

Different innovative synthesis and modification strategies have been developed to increase the efficiency of MIPs‘ performance. For example, and in some studies, structural-analogs have been used, as dummy templates for explosives (Table 1). Using dummy templates can help to prevent a bleeding effect after polymerization or even more effective formation of template-monomer complex before polymerization. The sizes of polymer particles have also been reduced to nanoscales and the porosity of the polymer matrix has been increased. These changes have helped increase the surface-area-to-volume ratio and improve the accessibility of the majority of imprinted cavities and, therefore, increase polymer capacity. This has also simplified the template removal and rebinding processes. In some other studies, polymerization, either on the surface or within the pores of a uniform nanoscale substrate, has also been used to produce more uniform and homogeneous MIP nanoparticles or MIP-modified nanoparticles (detailed MIP synthesis parameters of the published manuscripts are summarized in Table S1).

Most of the synthesized polymers were used in combination with sensor transducers. The majority of these sensors were either electrochemical or fluorescence sensors due to the fact that NACs can undergo stepwise reduction in an electrochemical reaction or quench the fluorescence emission of nearby fluorophores. An ideal modified sensor must be highly selective, sensitive, fast, robust, inexpensive, and have the ability to be miniaturized. To maximize the reproducibility, sensitivity and stability of MIP sensors, uniform and reproducible porous MIP nanolayers or nanoparticles, which are well adhered to the sensor’s surface, are required. It is worth noting that most MIP sensors were evaluated for the explosives in liquid samples. In comparison to liquid samples, the usage of MIP materials for gaseous samples is more limited due to the following reasons: (I) when MIP materials are used in the gas phase, the 3D shape of the recognition sites and spatial orientation of functional groups within them are altered, as the materials are dry. This causes the MIP to lose its selective recognition ability. (II) Most of the widely-used explosives (e.g., TNT) belong to a group of chemicals termed semi-volatile organic compounds with extremely low volatility. However, innovative strategies were also used to develop fluorescence [31] and integrated-optical-waveguide [39,40] sensors for TNT detection in air.

MIPs have also been used for sample preparation and separation purposes (analytical parameters of these methods are summarized in Table S2). They are used to pack columns and solid phase extraction (SPE) cartridges or as an extracting phase in the solid phase microextraction (SPME) method. Like sensors, sample preparation of explosives using MIPs is also largely limited to liquid samples and at ambient temperatures.

Valuable information for commercially available explosives detection methods has been gathered by the Center for Homeland Defense and Security (CHDS) [66], which enables us to have an overview and compare the developed methods throughout this review with those already available in the market. For trace detection of explosives (nitrates, chlorates, bromates, and peroxides)—vapor or particle—portable (1–6.6 kg) or bench-top (53–60 kg) detectors are available. They generally work on the basis of ion mobility spectrometry (IMS) [67], thermo-redox techniques or chemiluminescence, and fluorescence techniques or surface acoustic wave (SAW) method. They can mostly detect explosives between 1 ng to 100 ng as particles and 1 µg m^−3^ to 10 µg m^−3^ as vapor. These portable detectors mostly suffer from interferences and can detect nitrates without classification. However, the bench-top versions can identify many individual explosive compounds with little interference. The response times for all detectors are lower than 60 s. Additionally, they can operate in all expected environments and extreme environments, which must be considered as a critical point for newly developed ideas.

For determination of explosives in water samples, the federal facilities forum, a U.S. environmental protection agency (EPA) group, reported on the field sampling and selecting of on-site analytical methods [68]. In this paper, the authors gathered information from other manuscripts in which e.g., frequency of occurrence of specific explosives in groundwater was assessed by compiling analytical data on water samples collected from military installations. The obtained median levels for explosives using EPA Method 8330 (EPA 1998) were as following: TNT: (15 × 10^−9^ mol L^−1^), 1,3,5-TNB: (7 × 10^−9^ mol L^−1^), 2-amino-4,6-dinitrotoluene: (56 × 10^−9^ mol L^−1^), 2,4-DNT: (6.6 × 10^−9^ mol L^−1^), 4-amino-2,6-dinitrotoluene: (23 × 10^−9^ mol L^−1^), DNB: (4.6 × 10^−9^ mol L^−1^), Tetryl: (3.2 × 10^−9^ mol L^−1^), 2,6-DNT: (0.55 × 10^−9^ mol L^−1^), RDX: (13.5 × 10^−9^ mol L^−1^) and HMX: (257 × 10^−9^ mol L^−1^) [68]. In another study by the U.S. Army, the occurrence and concentration of explosive residues in groundwater were evaluated at open burning open detonation (OB/OD) sites. The obtained mean values for the detected explosives were as following: TNT: (141 × 10^−9^ mol L^−1^), RDX: (756 × 10^−9^ mol L^−1^), 2,4-DNT: (77 × 10^−9^ mol L^−1^), 2,6-DNT: (71 × 10^−9^ mol L^−1^), HMX: (1232 × 10^−9^ mol L^−1^); Tetryl was not detected. In both scenarios, TNT and RDX were detected in most evaluated sample sites. HMX was detected in a small number at a relatively high concentration.

## 2. Sensors

### 2.1. Electrochemical Sensors

Electrochemical sensors provide various cost-effective and simple platforms, which are relatively easy to modify to sense specific targets in liquid samples. When using electrochemical sensors, the redox activity of nitro groups associated with many explosives can be used for their detection [28]. Nitro groups (–NO_2_) can be reduced to hydroxylamine groups (–NHOH) under acidic conditions. Hydroxylamine groups can also be oxidized, when the applied potential is reversed towards positive voltages [24]. If these reactions occur on the surface of a modified electrode, which specifically binds explosives or explosive markers, the specific detection of these chemicals may be possible.

An electrochemical quartz crystal microbalance (EQCM) is a device that can be used simultaneously as an electrochemical transducer and a mass sensor to monitor either the current response or the mass-change on gold electrode as a result of an electrochemical reaction. Using electropolymerization, the gold electrode of an EQCM was modified with conductive MIP layers using the following template molecules: 2,4-dinitrotoluene (DNT), TNT, 1,3,5-trinitrobenzene (TNB) and 2,4,6-trinitrophenol (TNP) [22]. The modified sensors were then individually connected to a flow-injection-analysis system. For the reduction of each target, a specified negative potential was applied to the modified sensors. However, electrochemical decomposition of MIP layer occurred at −1.00 volt. The developed sensors provide suitable selectivity when the template of one MIP chemosensor was used to test the selectivity of other chemosensors. However, they suffer from a lack of sensitivity and have not been used for real samples (Table 2).

In another study, a commercially available screen printed electrode (SCPE) as an innovative miniaturized electrochemical platform was coated with a thick layer (200 µm) of TNT imprinted polymer [23]. Carboxylic acid (–COO^−^H^+^) groups in the backbone of the polymer were used as an electrolytic medium. Using cyclic voltammetry, reduction and oxidation peaks appeared for TNT using MIP-modified SCPE, while no peak was obtained with another SCPE, which was modified with non-imprinted polymer (NIP). This proved that recognition sites were imprinted within MIP, while NIP inhibited the access of TNT molecules to the working electrode. For quantification purposes, differential pulse voltammetry (DPV) was selected due to the easier identification of peaks at lower concentrations. The selectivity of the developed MIP-modified SCPE was evaluated using organic, anionic, and cationic targets. Among all evaluated organic molecules, only 2,4,6-trinitrobenzoic acid (TNBA) was detected, which has a similar structure to TNT. Additionally, the MIP-modified SCPE detected Cu^2+^ due to the cation-exchange nature of the polymer. However, the peak-potential of the Cu^2+^ did not interfere with that for TNT molecules. The results showed that the sensitivity of the bare SCPE and MIP-modified SCPE depends strongly on the acidity, ionic composition and concentration in the solution. After the working parameters were optimized, the sensitivity of the MIP-modified SCPE was higher than that of the MIP-modified EQCM [22] (Table 2). The authors also showed that their MIP-modified SCPE can be used for selective detection of TNT, even in 20 µL not-deaerated water sample. In general, commercially available SCPEs are cost-effective and disposable, and their related accessories are portable. These inherent advantages of bare SCPE can make their modified versions an ideal choice for field application. However, the obtained LOD for determination of TNT using the MIP-modified SCPE (0.50 × 10^−6^ mol L^−1^) is still higher than those mean values, as reported by EPA [68], for TNT concentration in groundwater samples in military installations (15 × 10^−9^ mol L^−1^) and groundwater samples in open burning open detonation (OB/OD) sites (141 × 10^−9^ mol L^−1^).

The surfaces of other known electrochemical electrodes e.g., carbon electrodes including carbon paste electrodes (CPEs) and glassy carbon electrodes (GCEs) and traditional gold electrodes have been modified with explosive-imprinted polymers using innovative strategies.

Alizadeh’s group introduced a simple and effective method to modify CPE material with imprinted polymer particles (synthesized by precipitation polymerization) for sensitive determination of TNT [24]. Later, they proposed a new variation [25] in which methacrylic acid-modified magnetic nanoparticles (Fe_3_O_4_ ≈ 22 nm) were coated with a TNT-imprinted organic nanolayer (≈100 nm). The modified magnetic particles were suspended in water samples and then collected and analyzed with a magnetic CPE. In both studies, the interference caused by various ions and molecules were examined. The results showed that the 5–7% relative-error of TNT signal happened when interference concentrations were greater than or equal to 30 times the TNT concentration. Both methods allowed sensitive determination of TNT in water samples (Table 2). However, as the former MIP-modified CPE proposed by Alizadeh [24] is easy to produce and can be regenerated by simply rubbing the used electrode on a paper, this strategy may prove to be more successful for the production of commercial sensors.

GCEs, which have lower porosity and higher mechanical rigidity than CPEs, have been also modified with explosives imprinted polymers using a range of different methodologies by different research groups [26,27,46,48,52].

Using a simple strategy, triacetone triperoxide (TATP)-imprinted sites were created among a uniform organic layer on the surface of GCE during electropolymerization of pyrrole [48]. The synthesized layer was shown to be selective towards TATP in the presence of a group of other explosives including pentaerythritol tetranitrate (PETN), TNT, 1,3,5-trinitro-1,3,5-triazine (RDX) and 1,3,5,7-tetranitro-1,3,5,7-tetrazocane (HMX). Although this sensor is easily prepared and shows a wide linear-range, it is less sensitive than the other modified GCEs presented in this review (Table 2).

Recently, Alizadeh et al. [46] reported a highly sensitive GCE modified with a mixture of RDX-imprinted polymer nanoparticles and multiwalled carbon nanotubes (MWCNTs). The combination of the unique electronic features of the MWCNTs and the selectivity and porosity of the MIP nano-spheres enabled sensitive (LOD: 20 × 10^−12^ mol L^−1^) and selective determination of RDX in water samples (Table 2). The obtained LOD could satisfactorily support RDX measurement in groundwater samples in military installations and in open burning open detonation (OB/OD) sites, which have, respectively, mean values as following: 13.5 × 10^−9^ mol L^−1^ and 756 × 10^−9^ mol L^−1^ (reported by EPA [68]). In this study, the preparation procedure is relatively simple and the sensor response towards RDX is 3.5 times higher than that of HMX and eight times more than TNT.

**Figure 1:**
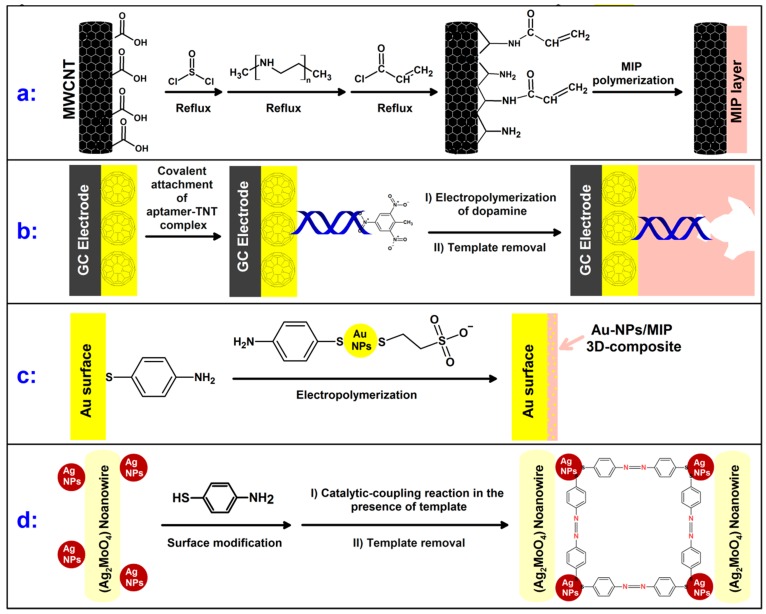
Developed synthesis strategies for explosive imprinted polymers used in electrochemical sensors (**a**, **b** and **c** in [52], [26] and [28], respectively), surface plasmon resonance sensors (**c** in [58,59,64]) and surface-enhanced Raman scattering sensor (**d** in [36]).

Using another strategy (Figure 1a), the surfaces of carboxylic acid functionalized MWCNTs were preliminary modified with a DNT imprinted polymer using a comparably complicated procedure [52]. MIP-modified MWCNTs were then mixed with a chitosan solution, which was dropped and dried on the surface of a GCE. The sensor response towards DNT was at least three times higher than that of the other analog chemicals, including TNT, TNB and 1,3-dinitrobenzene (DNB) each at 0.3 µM. In addition, the evaluated targets did not interfere with the DNT signal (at 0.1 µM), even when their concentrations were three times higher than the concentration of DNT. Despite the success of this method at selectively detecting the target molecule, the modification procedure is more complicated than the previous GCE method presented [46] and the sensitivity is not as high.

Nie et al. prepared a GCE modified with gold nanoparticles (AuNPs) and further coated it with a two-dimensional imprinted monolayer (2D-MIM) based on the self-assembled monolayers (SAMs) preparation procedure [27]. The 2D-MIMs can provide better site accessibility and lower mass-transfer resistance. Modified sensor for TNT provided a LOD (1.3 × 10^−8^ mol L^−1^), which enables its application for groundwater samples in open burning open detonation (OB/OD) sites, which contain a relatively high concentration of TNT (mean value: 14.1 × 10^−8^ mol L^−1^ [68]). The sensor’s selectivity was calculated by dividing the obtained peak currents (I_MIP_/I_NIP_) for TNT and its structural analogs each at 2.5 µM (TNT: 4.91, TNB: 1.36, DNT: 1.49 and DNB: 1.27). The optimized method was used successfully for the determination of TNT in spiked wastewater and river water samples. However, a 2D-MIMs modified sensor can also suffer from a lack of stability, as the film is not cross-linked and therefore this methodology may not be able to deliver a rugged sensor.

In another study, a complex procedure (Figure 1b) was used to modify a GCE with a layer of C60-AuNPs, which act as a porous conductive and biocompatible mediator. A further modification was made, whereby an amino-aptamer acted as the receptor within the imprinted cavity of the MIP layer, which was coated on the nanoparticles producing a hybrid receptor [26]. Despite the ultra-sensitivity obtained for the developed sensor (−1.148 × 10^12^ Ω mM^-1^ with LOD: 3.5 × 10^−18^ mol L^−1^), the preparation procedure is complicated. Furthermore, aptamers, which play a key role in the selectivity of this modified sensor, are inherently sensitive to harsh environmental conditions that restrict their comprehensive application. Besides, reported results, which are many orders of magnitude better than anything else in the field, may not promise a commercial product for field application.

AuNPs were also used by Riskin et al. [28] to coat a 4-aminothiophenol-modified gold electrode (Figure 1c). After the AuNPs were electropolymerized onto the surface of the electrode, they were joined together using bisaniline bridging units. Due to the π–π interaction of the bridging units with NACs, the modified sensor showed relative selectivity and sensitivity towards TNT in comparison to DNT, as TNT is a stronger π-acceptor. The authors showed that electropolymerization in the presence of picric acid, as a dummy template for TNT, could imprint the composite layer and therefore increase the adsorption capacity and sensitivity of the modified sensor due to the additional steric confinement of target molecules [28]. The sensor’s selectivity was also increased by about 10-fold after imprinting the composite layer. This method offers a modified sensor with high selectivity using a relatively simple methodology. Additionally, it provides a sensitive and fast sensor for determination of TNT in water samples (Table 2).

### 2.2. Fluorescence and Chemiluminescence Sensors

Nitrated explosives are able to quench the emission of nearby excited fluorophore species due to photoinduced electron transfer between the fluorophore and the nitrated explosive molecule [29,49]. MIP-modified fluorescence sensors can be prepared either by covalent attachment/physical encapsulation of quantum dots (QDs) or by using fluorophore functional-monomers.

Non-uniform TNT- and DNT-imprinted polymer microparticles (≤20 µm) were prepared using bulk polymerization [29]. Then, the carboxylic acid side groups of the MIP particles were activated to enable them to react covalently with the amine-functionalized CdSe quantum dots (CdSe QD-NH_2_) (λ_em_ 605 nm) in a zero-length cross-linking reaction. However, the selectivity of the developed sensors was not systematically evaluated. Despite the relatively simple methodology proposed, the limit-of-detections (LODs) obtained using this method were high (Table 2) due to the structure of the polymer particles.

Therefore, in a new study [53], a porous DNT-imprinted polymer layer was synthesized on the glass slide using bulk polymerization. The idea was to carry out the polymerization in the void spaces among silica particles (≈0.213 µm), which were coated on a glass slide. After polymerization, the silica particles were removed and the polymer surface was labeled with QD-NH_2_ using the same procedure described by Stringer et al. [29]. Although the LOD was improved by a factor of ten for DNT, the sample incubation time was increased from 10 min to 30 min (Table 2). The results showed that the increased porosity still could not provide a sufficiently sensitive fluorescence sensor. Further reduction in the size of polymer particle to the nanoscale could be investigated to improve the sensor’s sensitivity. For example, Zhang’s group [17,30] has proposed an interesting synthesis strategy to prepare MIPs that have polymer particles at the nanoscale. The idea was to carry out polymerization within the nanopores of a sacrificial-substrate to produce MIPs as nanowires or nanotubes for TNT. These materials have not been used in combination with a sensor or a sample preparation method, which can be evaluated in a new study.

**Figure 2:**
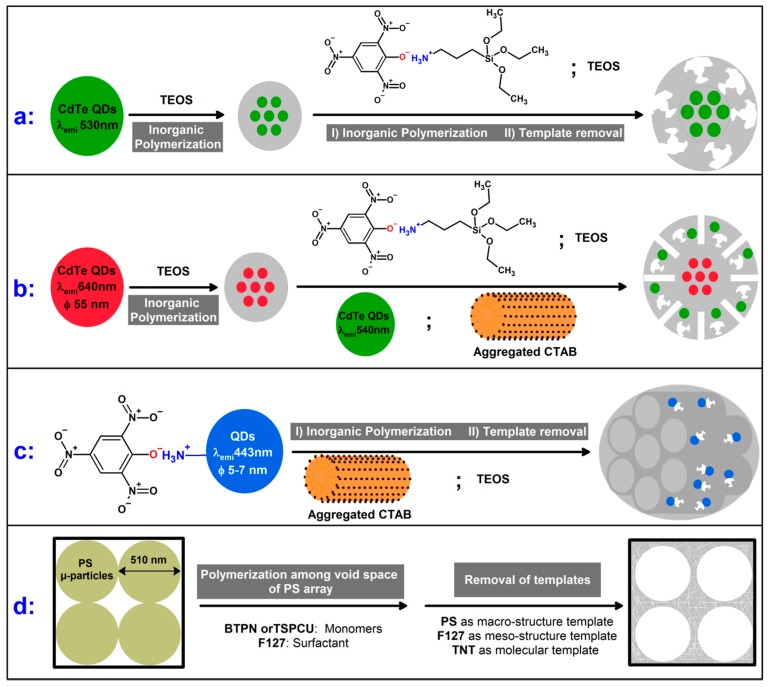
Developed synthesis strategies for explosive imprinted polymers used in fluorescence sensors (**a**, **b**, **c** and **d** in [61], [62], [63] and [31], respectively). TEOS: Tetraethyl orthosilicate; CTAB: Cetyltrimethylammonium bromide; PS: Polystyrene; BTPN: 1,5-Bis (allyloxy)naphthalene; TSPCU: Triethoxysilylated Coumarin, F 127: Pluronic F127.

Xu et al. [61] reported a fluorescence sensor in which CdTe QDs (λ_em_ 530 nm) were dispersed within a silica nanoparticle, which was subsequently covered with an imprinted silica layer using TNP as the dummy template for TNT (Figure 2a). Three synthesis methods (reverse-microemulsion, Stöber and seed-growth methods) were compared and the seed-growth method, which combined the advantages of the other two methods, produced QDs@MIP with the best fluorescence functionality. These modified particles were used for selective determination of TNT in soil samples. However, the sensitivity of the prepared sensor was weak due to the recognition-site’s poor accessibility and also the low probability that each recognition site is surrounded by QDs.

Two modifications to this sensor were proposed in a further article from Xu et al. [62]. Firstly, a mesoporous structure (pore diameters: 2–15 nm) was created in the imprinted shell layer using aggregated ionic surfactants as supramolecular templates in the co-condensation reaction. Secondly, the ratiometric fluorescence technique was used in which red CdTe QDs (λ_em_ 640 nm) were dispersed within the silica nanoparticle and green CdTe QDs (λ_em_ 540 nm) were dispersed in the mesoporous imprinted silica shell. The red CdTe QDs were not in direct contact with external TNT molecules and their wavelength was monitored as a reference signal (Figure 2b). Adding these two modifications, the detection limit of QDs@MIP sensors improved from µM [61] to nM [62]. In addition, a more reliable qualitative visual assessment of the sensor was also possible [62].

A further optimization of these sensors was performed [63], whereby the imprinting process was simplified. This was achieved by using the amine-functionalized QDs as the functional monomer. Cetyltrimethylammonium bromide (CTAB) was again used as the directing agent, to synthesize the previously described mesoporous polymer (Figure 2c). A further benefit of this change was that the optimized analysis time was reduced from 10 min to 4 min.

Mesoporous structures, this time in combination with macro- and microporous structures, were used to fabricate a fluorescence gas-sensor, which was used in a batch-mode for the fast (response time ≈ 120 s) determination of TNT molecules in gas phase [31]. In this study, polymerization of mesoporous organosilicas was performed in the void spaces of a close-packed, face-centered cubic arrangement of monodisperse polystyrene (PS) microspheres (diameter of each 510 nm). After the template removal (PS as the macro-structure template; aggregated ionic surfactants as the meso-structure template; TNT as the molecular template), TNT-imprinted recognition sites remained within the mesoporous structure in the macropore walls (Figure 2d). This hierarchically synthesized meso-macro-porous structure enabled fast and easy access to the imprinted cavities due to the higher surface areas and larger pore volumes [31]. Additionally, by replacing the fluorophore functional-monomer with a urea-functionalized dye, the selectivity of the synthesized porous layer was considerably improved. However, these materials have not been systematically evaluated to show their selectivity and sensitivity.

In contrast to all of the previously mentioned materials, which rely on a quenching mechanism to cause a change in the fluorescence, it has recently been shown [49] that the rigid polymer-matrix of MIP could facilitate fluorescence emission in the presence of nitrated explosive molecules. In this study, the surface of an indium-tin oxide glass-slide electrode was modified with TNP-imprinted polymer (TNP was used as template and target molecule) using electropolymerization. This polymer emitted a new fluorescence peak at ≈670 nm, when it was wetted with TNP solutions [49]. Compared to the evaluated interferences (TNT and phenol), the modified sensor showed the highest sensitivity towards TNP, which was five times more than that for TNT and six times more than that for phenol. However, it was not applied for a real sample measurement.

Another highly sensitive sensor was developed for the determination of TNT in water and soil samples using an indirect approach that measured the weak chemiluminescence (CL) emission of alkaline KMnO4/rhodamine B system [32]. In this study, ZnO QDs were synthesized and dispersed within TNT-imprinted silica nanoparticles using a similar approach to that of Xu et al. [61]. After template removal, the washed QDs@MIP nanoparticles catalytically amplified the weak CL emission. In the presence of TNT molecules, the imprinted nanoparticles lost their catalytic activity and the intensity of the CL was decreased. This reduction was used to calculate the TNT concentration [32]. Only one of the structural analogs (TNP) used for selectivity evaluation of the sensor caused a signal change (Signal_TNT_: ≈460 and Signal_TNP_: ≈60), showing that the sensor selectively detected TNT. Additionally, TNT could even be efficiently measured in the presence of a variety of different ions at high concentrations.

### 2.3. Surface Plasmon Resonance (SPR) and Localized Surface Plasmon Resonance (LSPR) Sensors

In SPR sensors, a thin metal layer is modified on one side (sensing side) and then irradiated from the other side (detection-side) with monochromatic light at different angles. When the incident light has sufficient momentum and strikes the metal surface at the correct angle of incidence, the oscillation mode of surface electrons can become coupled with that of the electromagnetic wave, causing the light to be absorbed. This specific resonance angle is sensitive to the changes in the surrounding environment of the sensing-side. As the adsorption of molecules on the sensing-side of the metal layer can shift the angle at which the light is absorbed, a suitably modified sensor could be used for the specific detection of a target molecule.

In the typical Kretschmann configuration, a prism is used to effectively couple the irradiated electromagnetic waves with the oscillation mode of the surface electrons in metal [69]. The prism was replaced with plastic optical fibers (POFs) [33,34] to reduce the size and cost of device, to enable remote sensing and to enhance the sensitivity of the sensor due to multiple interactions between the light and the detection side occurring in the optical fiber. One side of a POF was polished to produce a D-shaped POF and then coated successively with a photoresist buffer film, a thin gold layer, and a TNT-imprinted polymer layer [33]. The sensor showed low-sensitivity (Table 3), which also made it difficult to evaluate the selectivity of the modified MIP layer.

In order to increase the sensitivity of this SPR sensor, a D-shaped POF was covered directly with a composite layer containing a TNT-imprinted polymer and gold nanostars [34]. These metallic nanoparticles are of comparable or smaller size than the wavelength of incident light and therefore can confine and amplify the SPR, termed LSPR. The sensitivity of the LSPR sensor [34] (Table 3) was ≈3-fold that of the previously reported SPR sensor [33]. Additionally, it was shown that the sensitivity of the MIP-modified LSPR sensor can be further increased up to ≈10-fold when the substrate was tapered before modification [34]. Despite the increase in sensitivity as a result of this modification, these sensors still suffer from low sensitivity relative to the following SPR sensors.

Electronic coupling of LSPR to SPR was used to produce ultrasensitive, fast and selective SPR sensors for TNT [64], RDX [58], PETN [59], nitroglycerin (NG) [59] and ethylene glycol dinitrate (EGDN) [59] after a surface modification of the SPR gold surface with MIP/Au-NPs composite nano-layers (9.7 nm [64] and 10–12 nm [58]). These sensors were prepared by electropolymerization of AuNPs onto the surface of SPR sensors in the presence of the following dummy templates: picric acid as dummy template for TNT [64], Kemp’s Triacid for RDX [58], citric acid for PTEN and NG [59] and maleic acid or fumaric acid for EGDN [59]. Interestingly, citric acid as a dummy template could imprint the Au NPs/polymer composite layer for PETN, while succinic acid, fumaric acid and even the stereoisomer isocitric acid led to imprinted sites of poor affinity for this explosive [59], suggesting that the choice of dummy template is of vital importance.

### 2.4. Surface-Enhanced Raman Scattering (SERS)

In Raman scattering, light is inelastically scattered as it interacts with molecules. The energy change of the scattered light can be used as the structural fingerprint for the molecule under investigation. In SERS, Raman scattering is strongly enhanced when the target analytes are in proximity of metallic nanoparticles or SERS-active nanometer-scale patterning grid surfaces. The gold surface of Klarite, which is a commercial SERS-active substrate, was coated with a TNT-imprinted inorganic film (7–12 µm) using the sol-gel process to produce a selective sensor [35]. The sensor was neither fast nor sensitive (Table 3). This sensor was, however, stable for at least six months, which was presented as the highlight of this study.

To produce a sensitive SERS sensor with stable signals, a novel SERS substrate composed of silver nanoparticles coated on silver molybdate nanowires (Ag-SMNs) was introduced and modified with TNT-imprinted sites between the SERS-active nanowires [36] (Figure 1d). Using this new SERS substrate, the LOD was reduced from µM [35] to pM [36]. However, these sensors have not been used for TNT measurement in real samples.

### 2.5. Colorimetric Sensors

Colorimetric sensors are able to change their color in response to their surrounding environment. This type of visual detection is of great interest to the military and at security checkpoints due to the ease of reading and interpreting the signal. Photonic crystals (PCs) are biomimetic colorimetric substrates, which are composed of periodic optical nanostructures and reflect certain wavelengths of light depending on the angle of incident light, the average refractive index, and structural spacing of nanostructure constituents of PCs (Bragg-Snell law).

Lu et al. [37] synthesized monodisperse TNT-imprinted polymer particles (ø 210 nm) using emulsion polymerization. Then, the polymer particles were self-assembled on a glass slide to produce a three-dimensional (3D) close-packed film. Afterwards, an adhesive tape was pressed on the prepared film and the glass slide was removed. The color of the MIP-modified film on the adhesive tape changed from green to red in the presence of TNT molecules in an optimized solution (0→20 mM). Although this sensor was not sensitive, it showed high stability of up to three years.

Building on their previous work, an array of MIP-modified films on adhesive tapes in combination with principal component analysis was used to create “radar” patterns for detection of target compounds [38]. In this study, the same procedure was used to prepare a range of adhesive tapes modified with TNT-, DNT-, 2,6-dinitrotoluene (2,6-DNT)- and 4-nitrotoluene (4-NT)-imprinted polymer particles.

Although this idea could provide a promising avenue to produce detection kits for preliminary identification of explosives, these sensors do not provide a fast response and still suffer from low sensitivity.

### 2.6. Integrated-Optical-Waveguide (IOW)

Attenuation or phase change of a propagated wave through a cylindrical or planer substrate with total internal reflection characteristics depends on the near-surface composition of the substrate. When the thickness of the substrate is reduced to the dimensions of propagated wave, the substrate is termed an IOW and sensors made using IOWs show greater sensitivity than the thicker substrate.

Edmiston’s group has studied the effectiveness of sensors using IOWs both for TNT detection in air in relation to attenuation and phase shift. In one study, a planar IOW substrate (SiO_2_/TiO_2_ glass layer, thickness 0.8–1.0 µm) was coated with TNT-imprinted silica layer (0.3–1.0 µm) using dip-coating in sol solution [39]. Using the same coating method, a waveguide interferometer was coated with TNT-imprinted silica layer (0.2, 36 nm) [40]. Attenuation of total reflection (ATR) at 530 nm [39] and phase shift for 633 nm [40] were used for the sensitive determination of vapor-phase TNT (Table 3). Using these sensors in the gas phase, TNT molecules were attached irreversibly to the sensor, which was presented as an advantage when using prolonged sampling times and for extremely low concentrations [39,40]. The modified sensors responded suitably, even at high relative-humidity up to 50% [39] and 55% [40]. In addition, high concentrations of volatile compounds (which were emitted from the gasoline and cologne containers) did not affect the TNT detection, although they slowed the response time [39].

### 2.7. Quartz Crystal Microbalance (QCM)

In order to detect TNT molecules in the gas phase, two QCM sensors were coated with MIP layers by UV polymerization [41,42]. Different MIP layer thicknesses were obtained, depending on the covering methods as follows: 20–500 nm by manual coating method, 3300 nm by automated nano-plotter and 500–3000 nm via spin coating. Although little evidence regarding either the selectivity or sensitivity of the sensors was presented, the major drawback of this approach was the long response time. As TNT detection in the gas phase is challenging, the idea of a MIP-modified QCM may still prove effective if further developed e.g., by using porous nanostructured MIPs.

For liquid samples, the gold surface of a QCM was modified with a two-dimensional molecularly imprinted monolayer (2D-MIM) [54], using a very similar approach described by Nie et al. [27]. In this study [54], butanethiol in ethanol was used to prepare a self-assembled monolayer in the presence of DNT molecules. In general, a 2D-MIM modified sensor suffers from a lack of stability. Additionally, this modified QCM sensor is neither fast nor sensitive (Table 3) and does not discriminate against molecules with smaller size than the template (e.g., toluene and 4-NT).

## 3. Sample Preparation

There is a great variety of sorbent-based extraction methods that have been developed for the extraction of chemical compounds from liquid samples. However, explosive-imprinted polymers have, so far, only been used either to pack solid phase extraction (SPE) cartridges and columns or to perform SPE in batch mode.

Lordel et al. optimized a synthesis protocol for a DNT-imprinted polymer using sol-gel polymerization with a template/monomer/crosslinker ratio at 1/1/5 [55]. Fifty mg of the synthesized polymer particles (25–36 nm) were used to fill a 1 mL SPE cartridge. Using an optimized extraction procedure, the cartridge selectively extracted DNT and TNT from spiked simulated post-blast samples. Although this method recovered DNT (80%) and 2,6-DNT (68%) from 15 mL of a spiked water sample with relatively high percentages, these values for TNT (46%) and tetryl (29%) were low. Therefore, new sol-gel imprinted polymers were synthesized using the same precursors, but at different template/monomer/crosslinker ratios: 1/4/20 and 1/4/30 [56]. SPE cartridges, packed with the new synthesized polymers, recovered more than 79% of all the targets. Building on the previous work, the polymer synthesized at 1/4/30 ratio was used to pack a pre-column and then connected online with a reversed-phase LC system [57]. This online system increased the extraction recoveries to more than 90%.

Xue’s group optimized a synthesis protocol with hexanitrohexaazaisowurtzitane (CL-20) as the template molecule using precipitation polymerization [50]. One hundred mg of the optimized MIP was packed in a 1 mL SPE cartridge and successfully used to selectively extract CL-20 from soil samples. The same group further developed this idea using a new synthesis strategy to increase the porosity of the CL-20 imprinted polymers [51]. In this new study, a modified silica particle was first coated with a MIP shell before being removed using hydrofluoric acid to produce CL-20 imprinted hollow spheres (with an outer diameter of 0.25–0.5 µm and a thickness of 30–100 nm). Again, a SPE cartridge packed with MIP sorbent was used, but this time for the extraction of a group of explosives (CL-20, TNT, RDX and HMX) from simulated post-blast samples prepared from motor oil and vacuum pump oil.

In another study by Xue’s group, 14 different imprinted polymers were synthesized in the presence of either HMX or RDX as template molecules using precipitation polymerization [47]. RDX is the major byproduct of HMX synthesis and their separation is difficult due to their similar sizes and physical properties. However, a SPE cartridge packed with the optimized MIP could satisfactorily separate them using a flash-chromatography procedure.

In separation science, band-broadening is a major drawback that inversely affects separation efficiency. Trammel et al. [43] synthesized two series of TNT-imprinted periodic mesoporous organosilicas using two different bissilylated organic precursors [43]. They showed that columns which are packed with sorbents with a narrower pore size distribution released the adsorbed targets over a much shorter time.

Ebrahimzadeh et al. showed the advantage of coupling MIP-SPE with other techniques. A batch-mode SPE was coupled with a dispersive-liquid-liquid-microextraction to allow the selective extraction of 3-nitrotoluene (3-NT), whilst increasing the total pre-concentration factor by up to 2800 [65]. In this study, final extracts were analyzed using gas chromatography with a flame ionization detector. Ion mobility spectrometry, a known handheld and portable analytical technique, has also been used to analyze the extracts obtained from a batch-mode SPE [44].

Solid phase microextraction (SPME) is a non-exhaustive sampling and sample preparation method, which has the potential for field application. However, there is just one example where MIP-SPME was developed for use with gaseous samples [45]. In this study, TNT-imprinted polymer powder was coated on a silica support of a SPME fiber using epoxy resin glue. The modified SPME fiber was used to extract TNT molecules from the headspace of a heated sample. The main drawback is the prolonged sampling time (40 min), which is a known difficulty when working with gaseous samples.

## 4. Conclusions and Future Perspectives

This paper reviews the developments in the synthesis and use of selectively imprinted polymers for explosives. MIPs are promising artificial selective sorbents, which can be used in a wide range of conditions; they are relatively stable, simple to produce, and easy to modify for a variety of target molecules. Despite the variety of different approaches in the scientific literature, there are currently no commercially available MIP-modified sensors or sample preparation methods for explosives in liquid samples. For targets in gaseous samples, their implementation is even more challenging, due to changes in the conformation of the binding sites when the polymer is dried. Novel synthesis methods are required to overcome these limitations. New sampling methodologies (e.g., spraying micro-size organic droplets which are loaded with MIP nanoparticles into the gas samples [70]) could help to accelerate the selective and efficient extraction of targets from gas samples. In the future, new, sensitive, low-cost and disposable sensor substrates (e.g., split-ring resonators [71,72]) could be modified with nanoscale MIP materials, as nanolayers or nanoparticles, to create a commercial sensor for explosives.

## Figures and Tables

**Table 1:** Explosives and explosive markers used as template molecules.

Chemicals	Abbreviation	Name	Application	Used in
Explosive	TNT	2,4,6-Trinitrotoluene	Secondary explosive (dumped)	[13,14,15,16,17,18,19,20,21,22,23,24,25,26,27,28,29,30,31,32,33,34,35,36,37,38,39,40,41,42,43,44,45]
	RDX	1,3,5-Trinitro-1,3,5-triazine	Secondary explosive (dumped)	[46,47]
	PETN	Pentaerythritol tetranitrate	Secondary explosive (boosters)	-
	NG	Nitroglycerin	Propellants, smokeless powders ingredient	-
	EGDN	Ethylene glycol dinitrate	Propellants, smokeless powders ingredient	-
	HMX	1,3,5,7-Tetranitro-1,3,5,7-tetrazocane	Secondary explosive	[47]
	TATP	Triacetone triperoxide	Primary explosive	[48]
	1,3,5-TNB	1,3,5-Trinitrobenzene	Explosive and also Biological degradation product of TNT	[22]
	TNP or Picric acid	2,4,6-Trinitrophenol	Explosive	[22,49]
	Tetryl	*N*-methyl-*N*-2,4,6-trinitroaniline	Secondary explosive (boosters)	-
	CL-20	Hexanitrohexaazaisowurtzitane	Rocket propellants	[50,51]
	HMTD	Hexamethylene triperoxide diamine	primary explosive	-
Explosive marker	DNT	2,4-Dinitrotoluene	It is a precursor to trinitrotoluene and can be used as an explosive marker. DNT is environmentally more stable than 1,3-DNB	[16,21,22,29,38,52,53,54,55,56,57]
	1,3-DNB	1,3-Dinitrobenzene	It is a precursor to trinitrotoluene and can be used as an explosive marker.	-
	4-amino-2,6-DNT	4-Amino-2,6-dinitrotoluene	Reduction product of TNT due to degredation	-
Dummy template	Kemp’s Triacid	Cis,cis-1,3,5-Trimethyl-1,3,5-cyclohexanetricarboxylic acid	It is used as dummy template for RDX.	[58]
	Citric acid	2-Hydroxypropane-1,2,3-tricarboxylic acid	It is used as dummy template for PETN and NG.	[59]
	Maleic acid	Cis-butenedioic acid	It is used as dummy template for EGDN.	[59]
	Fumaric acid	Trans-butenedioic acid	It is used as dummy template for EGDN.	[59]
	TNP or Picric acid	2,4,6-Trinitrophenol	It is used as dummy template for TNT.	[60,61,62,63,64]
	2,6-DNT	2,6-Dinitrotoluene	-	[38]
	4-NT	4-Nitrotoluene	-	[38]
	3-NT	3-Nitrotoluene	-	[65]

**Table 2:** Analytical figures of merit for developed MIP-modified electrochemical, fluorescence and chemiluminescence sensors.

Target	LOD (mol L^−1^)	Sensitivity	Linear Range (mol L^−1^)	Analysis Time	Sensor	Ref.
TNP	CA: 0.69 × 10^−3^ PM:0.02 × 10^−3^	CA: 7.34 µA mM^−1^ PM: 27.3 Hz mM^−1^	(0.7–5.6) × 10^−3^	10 min	EQCM	[22]
TNT	CA: 0.62 × 10^−3^ PM: 0.07 × 10^−3^	CA: 5.65 µA mM^−1^ PM: 21.4 Hz mM^−1^				
TNB	CA: 0.27 × 10^−3^ PM: 0.15 × 10^−3^	CA: 6.33 µA mM^−1^ PM: 8.6 Hz mM^−1^				
DNT	PM: 0.76 × 10^−3^	PM: 1.3 Hz mM^−1^				
TNT	0.50 × 10^−6^	25-200 µA mM^−1^	-	≈3 min	SCPE	[23]
TNT	1.5 × 10^−9^	1.33 × 10^4^ µA mM^−1^	(0.005–1) × 10^−6^	≈11 min	CPE	[24]
TNT	0.5 × 10^−9^	4.423 × 10^4^ µA mM^−1^	(0.001–0.13) × 10^−6^	≈5 min	CPE	[25]
TATP	0.36 × 10^−6^	7.25 × 10^1^ µA mM^−1^	(0.37–199) × 10^−6^	-	GCE	[48]
RDX	20 × 10^−12^	7.1 × 10^6^ µA mM^−1^	(0.1–10) × 10^−9^	≈15 min	GCE	[46]
DNT	1.0 × 10^−9^	0.6 × 10^4^ µA mM^−1^	(0.0022–1) × 10^−6^	≈11 min	GCE	[52]
TNT	1.3 × 10^−8^	3.0205 × 10^2^ µA mM^−1^	(0.04–3.2) × 10^−6^	30 s	Modified GCE	[27]
TNT	3.5 × 10^−18^	−1.148 × 10^12^ Ω mM^−1^	(0.01–10000) × 10^−15^	≈35 min	Modified GCE	[26]
TNT	2.0 × 10^−10^	≈6.1 × 10^3^ µA mM^-1^	-	≈1.5 min	Modified gold electrode	[28]
TNT DNT	TNT: 4.07 × 10^−5^ DNT: 3.01 × 10^−5^	-	-	TNT: 1 min DNT: 10 min	QD-MIP particle	[29]
DNT	3.01 × 10^−6^	^a^ ≈20.27 mM^−^^1^	(5.5–82.4) × 10^−6^	≈30 min	QD-MIP porous film	[53]
TNT	0.28 × 10^−6^	^a^ ≈61.2 mM^−^^1^	(0.8–30) × 10^−6^	≈10 min	QDs@MIP	[61]
TNT	1.5 × 10^−8^	^a^ ≈1818 mM^−^^1^	(5–60) × 10^−8^	≈10 min	Red-QDs@ green-QDs/MIP	[62]
TNT	1.7 × 10^−8^	^a^ 940 mM^−^^1^	(5–200) × 10^−8^	4 min	QD-NH_2_-MIP	[63]
TNP	0.87 × 10^−12^	13.7 × 10^6^ mM^−1^	(0.87–89) × 10^−12^	-	MIP-modified ITO electrode	[49]
TNT	30 × 10^−12^	2.16 × 10^6^ mM^−1^	(8.81–22000) × 10^−11^	≈18 s	Imprinted QDs@SiO_2_ act as catalyzer	[32]

^a^ K_sv_ in Stern-Volmer plot; CA: Chronoamperometry; PM: Piezoelectric microgravimetry.

**Table 3:** Analytical figures of merit for developed MIP-modified SPR, LSPR, SERS, IOW and QCM sensors.

Target	LOD (mol L^−1^)	Sensitivity	Linear Range (mol L^−1^)	Analysis Time	Sensor	Ref.
TNT	51 × 10^−6^	27 nm mM^−1^	(83–130) × 10^−6^	5 min	SPR/MIP layer-Gold layer-POF	[33]
TNT	GNS-MIP/ POF: 2.4 × 10^−6^ GNS-MIP/tapered POF: 0.72 × 10^−6^	^b^ GNS-MIP/ POF: 85 nm mM^−1^; ^b^ GNS-MIP/tapered POF: 830 nm mM^−1^	-	5 min	LSPR/MIP_GNC layer-POF	[34]
TNT	10 × 10^−15^	^C^ ≈1.2 × 10^12^ mM^-1^	(10–100) × 10^−15^	≈15 s	SPR-LSPR/Gold layer-Prism	[64]
RDX	12 × 10^−15^	^C^ ≈0.4 × 10^12^ mM^−1^	≈(12–300) × 10^−15^	≈15 s	SPR-LSPR/Gold layer-Prism	[58]
PETN NG EGDN	PETN: 200 × 10^−15^ NG: 20 × 10^−12^ EGDN: 400 × 10^−15^	PETN: ^C^ ≈7.4 × 10^9^ mM^−1^ NG: ^C^ ≈0.071 × 10^9^ mM^−1^ EGDN: ^C^ ≈3.5 × 10^9^ mM^−1^	P: ≈(0.2–8) × 10^−12^ N: ≈(20–400) × 10^−12^ E: ≈(0.2–5) × 10^−12^	≈15 s	SPR-LSPR/Gold layer-Prism	[59]
TNT	3 × 10^−6^	^D^ ≈4 × 10^4^ mM^−1^	≈(?–5) × 10^−5^	Incubation time: 24 h	SERS	[35]
TNT	1 × 10^−12^	-	(1–10000) × 10^−11^	≈ 60 s	SERS	[36]
TNT	^e^ 5 ppb V	0.13 × 10^12^ mM^−1^	^e^ (4–10) ppb V	100 s	IOW	[39]
TNT	^e^ 2.4 ppt V	8 × 10^-4^ (ppt V) ^−1^	^e^ (20–140) ppt V	120 s	IOW	[40]
DNT	-	≈900 Hz mM^−1^	(20–100) × 10^−6^	≈200 min	QCM	[54]

^b^ Sensogram was obtained using the obtained shift for resonance angle (nm) in different TNT concentration (mM); ^C^ Sensogram was obtained using the obtained changes in the reflective intensity of resonance angle at TNT: 0 mM (a.u.) by adding different TNT concentration (mM); ^D^ The obtained signal was the height of spectral band resulting from the nitrate stretching band at 1352 cm^−1^; ^e^ Gas-sensors; GNS: Gold nanostars; POF: Plastic optical fiber.

## Supplementary Materials

The following are available online at https://www.mdpi.com/2073-4360/11/5/888/s1, Table S1. Detailed synthesis-parameters used for MIPs synthesis in published manuscripts. Table S2: Analytical parameters of sample preparation methods for liquid and gaseous samples

## Abbreviations

Ag-SMNs	Silver molybdate nanowires
CPE	Carbon paste electrode
EQCM	Electrochemical quartz crystal microbalance
GCE	Glassy carbon electrode
IOW	Integrated-Optical-Waveguide
LSPR	Localized surface plasmon resonance
NACs	Nitroaromatic compounds
2D-MIMs	Two-dimensional molecularly imprinted monolayers
PS	Polystyrene
PCs	Photonic crystals
POFs	plastic optical fibers
SPR	Surface plasmon resonance
SERS	Surface-enhanced Raman scattering
