# Supplementary material for: Molecularly Imprinted Polymer Materials as Selective Recognition Sorbents for Explosives: A Review
Source: Polymers (Basel). 2019 May 15;11(5):888. doi: 10.3390/polym11050888 (PMC6572358; doi:10.3390/polym11050888)
Supplement: Supplementary file 1 [file polymers-11-00888-s001.pdf]

polymers

Supplementary Materials: Molecularly Imprinted Polymer Materials as Selective Recognition Sorbents for Explosives: A Review

Mashaalah Zarejousheghani, Wilhelm Lorenz, Paula Vanninen, Taher Alizadeh, Malcolm Cämmerer and Helko Borsdorf

Table S1. Detailed synthesis-parameters used for MIPs synthesis in published manuscripts.

Used technique	Surface	Surface modifier	Template (mmole)	Monomer (mmole)	Cross-linker (mmole)	Initiator (mg)	Porogen (mL)	Non-porogen solvent Or extra additive	MIP format	Application	Ref.
Organic polymerization/Surface modification	Alumina (porous membrane)	APTS	TNT (20)	AAm (80)	EGDMA (480)	AIBN (50)	Acetonitrile (30)	-	• Nanowire<br>• Nanotube<br>(polymer mixture ratios were changed)	-	[1]
Inorganic polymerization/Surface modification	Alumina	APTS	TNT (0.1)	APTS (0.21)	TEOS (1.13)	pH:5.1 and then Temperature	Acetonitrile (0.2 mL)	Under stirring, 4 mL ethanol, 1.13 mmole TEOS, 50 mg of CTAB, and 0.25 mL of sodium acetate buffer (pH, 5.1) were orderly added to the polymer mixture	• Silica nanotube	-	[2]
Organic polymerization/Suspension polymerization	-	-	TNT or 2,4-DNT (2)	AAm and MAA for TNT/ MAAm for 2,4-DNT (8)	EGDMA (50)	AIBN (200)	Chloroform (25)	Under stirring, the polymer mixture was added to 120 mL water containing PVA (mean Mw 22,000)	• Heterogeneous polymer beads	-	[3]
Inorganic-organic polymerization/Surface coating using dip-coating method	Microscope slides [4] / silicon wafers [5]	-	In a semi-covalent approach, 4-methyl-3,5-dinitrobenzyl alcohol was reacted with 3-isocyanatopropyltrimethoxysilane (7.5×10^{-3})		BTEB/TMSE-Pyr [4]/ BTB [5] (7.5×10^{-1})	Prolonged time and then temperature	THF or acetonitrile (50)	81 µL of H_2O premixed with 75 µL of 1M TBAF in THF	• Transparent monolithic films	-	[4,5]
Organic polymerization/ Electropolymerization on the modified gold electrode surfaces	Planner gold surface	4-aminothiophenol	Picric acid (as dummy template for TNT) [6,7], Kemp's Triacid (as dummy template for RDX) [8], citric acid (as dummy template for PETN and NG) and maleic acid or fumaric acid (as dummy template for EGDN) [9]	Thioaniline/ mercaptoethane sulfonic acid-capped Au NPs (3.5 nm) (1 mg mL^{-1})	-	-	0.1 M phosphate buffer pH = 7.4	-	• MIP/Au nanoparticle composite layer	Environmental water samples [6]	[6–9]
Organic polymerization/bulk polymerization	-	-	TNT or 2,4-DNT (0.025)	MAA (1.0)	EGDMA (4)	HCPK (10.2)	Chloroform 50% (v/v)	-	• Not-uniform particles	-	[10]
Organic polymerization/precipitation polymerization	-	-	TNT (1.0)	MAA (4.0)	EGDMA (24)	AIBN (32.8)	Chloroform (50)	-	• Heterogeneous polymer beads	Water and soil samples	[11]
Organic polymerization/Surface modification	PMMA (the core of side-polished POFs)	-	TNT (0.09)	MAA (0.35)	EGDMA (3.5)	AIBN (15)	-	GNS (mg) was mixed with mL prepolymeric mixture (0.67 mg Au/mL)	• MIP/GNS composite layer	-	[12]

Organic polymerization/Surface modification	Gold layer		TNT (0.09)	MAA (0.35)	EGDMA (3.5)	AIBN (15)	-	-	• MIP uniform layer	-	[13]
Organic polymerization/Surface modification	Silver nanoparticles coated on silver molybdate nanowires	4-aminothiophenol	TNT	4-aminothiophenol (surface modifier)	p,p′-dimercaptoazobenzene (monomer react with each other to produce it.)	photonic catalytic coupling	-	-	• Imprinted sites were prepared among aggregated nanoparticles.	-	[14]
Inorganic-organic polymerization/Surface coating using spin coating	Klarite substrate (A SERS-active surface)	MPTMS was used as one of precursors to connect the MIP film to gold surface	TNT (1.48×10^{-3})	APTS (1.51×10^{-2})	TriEOS (0.552)	Prolonged time (2-3 days) at room temperature	Ethanol (1.25) Acetonitrile (0.15)	MPTMS (1.51×10^{-2} mmol) HCL (6.25 µL)	• Transparent monolithic films	-	[15]
Organic polymerization/Surface modification	Fun-MWCNTs/PEI	Functional groups containing –C=C And –NH2 groups	2,4-DNT (0.1)	–NH2 group at the MWCNTS (??) Acrylamide (0.5)	EGDMA (2.5)	AIBN (15)	Acetonitrile (15) Toluene (2.5) *N,N*-dimethylformamide (3)	-	MIP thin layer at the surface of MWCNs	Water sample	[16]
Organic polymerization/Surface modification	magnetic nanoparticles (22 nm)	MAA	TNT (1)	MAA (4)	EGDMA (20)	AIBN (50)	Acetonitrile (30)	-	•Core-shell (Fe3O4/MIP layer; the shell thickness was tunable by changing the total amount of functional monomers and cross-linking agents)	Real Water samples	[17]
Organic polymerization/Surface modification	Screen printed electrode	-	TNT 20 mg	MAA (30 µL)	EGDMA (665 µL)	AIBN (15)	-	-	• MIP uniform thick layer	-	[18]
Organic polymerization/ Electropolymerization on the modified GCE	Glassy carbon electrode	C_{60} and Au NPs	TNT/amino-Aptamer complex	Dopamine (5)		-	10 mM phosphate buffer pH=7.4	-	Composite C60/Au NPs/aptamer/polydopamine layer	Soil and water samples	[19]
Organic polymerization/ Electropolymerization on the polished GCE	Glassy carbon electrode	-	TATP (100)	Pyrrole (100)		-	0.1 M LiClO4 supporting electrolyte	-	Conductive polypyrrole film	-	[20]
Organic polymerization/ Surface modification using Electropolymerization	Gold layer	-	Four different MIP layers for 2,4,DNT-TNT-1,3,5-TNB, Picric acid (0.5)	For each polymer layer Bis(2,2′-bithienyl)-(4-aminophenyl)methane (0.5)	tris([2,2′-bithiophen]-5-yl)methane (1.5)	-	0.1 M (TBA)ClO_4 in ACN/ DCB (1:1, v:v),	-	Conductive polythiophene film	-	[21]
Organic polymerization/Surface modification	Glass substrate	Polystyrene crystal array	TNT (4.4×10^{-4})	BTPN (8.8×10^{-4})	BTME (1)	HCL 12.3 µL Prolonged time (8 days at 60°C	EtOH (2.93) Water (0.38 mL)	0.019 mmoL F127 as as meso-structure-directing agent	Highly porous film containing macro-, meso- and micro-structures	-	[22]
Organic polymerization/Surface modification	Glass substrate	Polystyrene crystal array	TNT (2.84×10^{-3})	TSPCU (9.48×10^{-4})	TEOS (1)	HCL 105 µL Prolonged time (8 days at 60°C	EtOH (2.49) Water (0.73 mL)	0.0226 mmoL F127 as as meso-structure-directing agent	Highly porous film containing macro-, meso- and micro-structures	-	[22]
Inorganic silica core containing QDs-imprinted silica shell (polymerization using reverse-microemulsion method)	-	-	TNP (5 mg)	APTS (20 µL)	TEOS (100 µL)	60 µL ammonia and about 24 h stirring	n-hexanol (1.8 mL); cyclohexane (7.7 mL)	Cd Te QDs (500µL); TX-100 (1.8 mL); PDDA (60 µL)	Uniform core-shell particles (50 nm)	-	[23]

Inorganic silica core containing QDs-imprinted silica shell (polymerization usin Stöber method)	-	-	TNP (100 mg)	APTS (250 µL)	TEOS (1 mL)	1 mL ammonia and about 24 h stirring	Ethanol (20 mL)	Cd Te QDs (5mL);	Uniform core-shell particles (50 nm)	-	[23]
Inorganic silica core containing QDs- imprinted silica shell (polymerization using seed-growth method)	-	-	TNP (100 mg)	APTS (250 µL)	TEOS (1 mL)	1 mL ammonia and about 24 h stirring	Ethanol (20 mL)	QDs@MIP seeds (50nm) prepared by reverse-microemulsion method (50mg); Cd Te QDs (5mL);	Uniform core-shell particles (200 nm)	Soil samples	[23]
Inorganic silica core containing red QDs-imprinted mesoporous silica shell containing green QDs (polymerization using seed-growth method)	Red QDs@SiO2 (10 mg)	-	TNP (5 mg)	APTS (20 µL)	TEOS (100 µL)	0.1 mL NaOH (0.2 M) and about 24 h stirring	Water (17 mL) Ethanol (0.2 mL)	2 mL green QDs solution; 0.8 mL CTAB solution (0.2 M)	Uniform core (55 nm)-shell (35 nm) particles	Soil and tap water	[24]
Organic polymerization/ Surface modification using Electropolymerization	ITO electrode	-	TNP (0.3mM)	NH2-S4 (as main monomer; 0.3mM) CLM (as crosslinker and functional monomer; 0.6 mM)		-	0.1 M (TBA)ClO4 in ACN/ Toluene (95:5, v:v),	-	Conductive film which has fluorescence emission in the presence of TNP	-	[25]
Organic bulk polymerization	Glass substrate	Silica NPs crystal array	2,4-DNT (0.02)	acrylic acid (4.4 mM) methyl acrylate (5.8 mM)	EGDMA (2.9 mM)	AIBN (6 mg))	EtOH (0.6 mL)	-	Porous film after removing the silica NPs in HF	-	[26]
Inorganic mesoporous silica particles containing QDs inside the wall od pores	-	-	TNP (10 mg)	Amino-QDs (2 mL)	TEOS (1mL)	NaOH 2M (0.7 mL) 70°C for 3 h	2 mL of ? And 100 mL of ?	100 mL CTAB solution (0.0055) M)	Not-uniform mesoporous silica particles	Soil and water	[27]
Self-assembly of alkanethiol monolayer on the surface of gold in the presence of template molecules	Gold surface	-	2,4-DNT (5 mM)	Butanethiol 1% w/v	-	-	Ethanol	-	A uniform monolayer (thickness 100 nm)	-	[28]
Inorganic polymerization/Surface modification	Polystyrene (PS) particles (220 nm) (14 mg)	Carboxyl groups	TNT (0.2 mmol)	APTS (0.4 mmol)	TEOS (2 mmol)	Sodium acetate buffer (pH 5.1; 1.25 mL)	Acetonitrile (20 mL)	-	Core-shell (PS/imprinted silica layer)	-	[29]
Inorganic polymerization/imprinted silica nanoparticles contained QDs	-	-	TNT (50 mg)	APTS (500 µL)	TEOS (2 mL)	8% NH4OH (2 mL) And 16 h stirring	Acetonitrile (20 mL)	APTS-modified ZnO QDs (600 mg)	Spherical particles (15-90 nm)	Soil and water	[30]
Inorganic polymerization/sol-gel method	-	-	2,4-DNT (0.5 mmol)	PTMS (0.5 mmol)	TEOS (2.4 mmol)	200 µL ammonia and heating at 40 °C for 24 h and then heating at 120 °C for 18 h	1 mL water	-	After crushing and sieving (regular particles 25-36 nm)	Water samples (Simulated sample from motor oil)	[31]
Organic polymerization/bulk method	-	-	3-NT (4 mmol)	MAA (16 mmol)	EGDMA (80 mmol)	AIBN (200 mg)	DCM (50 mL)	-	After crushing and sieving (regular particles 50-80 nm)	Wastewater	[32]
Organic polymerization/precipitation method	-	-	TNT (0.5 mmol)	Acrylamide (AM) (3 mmol)	EGDMA (3 mmol)	ABVN (0.18 mmol)	Acetonitrile (40 mL)	-	Inhomogeneous spherical particles	industrial waste water and surface water	[33]
Organic polymerization/Surface modification	Silica particles	KH-570	CL-20 (1.6 mmol)	Acrylamide (AM) (9.6 mmol)	EGDMA (38.4 mmol)	ABVN (0.62 mmol)	Acetonitrile (200 mL)	1.5 g of modified silica particles	The silica particles were removed by HF after polymerization to produce	simulated post-blast samples	[34]

									hollow microspheres (0.25–0.5 μm) (and thickness of wall: 30–100 nm)	prepared from motor oil and vacuum pump oil	
Organic polymerization/precipitation method	-	-	HMX (3 mmol)	MAA (24 mmol)	TRIM (24 mmol)	AIBN (50 mg)	Acetonitrile (200 mL)	-	irregular aggregated particles (5–15 μm)	-	[35]
Organic polymerization/precipitation method	-	-	HMX (3 mmol)	Acrylamide (24 mmol)	TRIM (72 mmol)	AIBN (50 mg)	Acetonitrile (200 mL)	-	Inhomogeneous microspheres (0.5–5 μm)	-	[35]
Organic polymerization/precipitation method	-	-	CL-20 (0.25 mmol)	Acrylamide (1.5 mmol)	EGDMA (6 mmol)	AIBN (5 mg)	Acetonitrile (20 mL)	-	Irregular microparticles	Soil samples	[36]
Inorganic polymerization/sol-gel method	-	-	2,4-DNT (0.5 mmol)	PTMS (2 mmol)	TEOS (10 mmol)	800 μL ammonia and heating at 40 °C for 24 h and then heating at 120 °C for 18 h	Water (4 mL)	-	After crushing and sieving (regular particles 25–36 nm)	Water samples (Simulated sample from motor oil)	[37]
Inorganic polymerization/sol-gel method	-	-	2,4-DNT (0.25 mmol)	PTMS (1 mmol)	TEOS (7.5 mmol)	565 μL ammonia and heating at 40 °C for 24 h and then heating at 120 °C for 18 h	Water (2.85 mL)	-	After crushing and sieving (regular particles 25–36 nm)	Water samples (Simulated sample from motor oil)	[37]

Table S2. Analytical parameters of sample preparation methods for liquid and gaseous samples.

		Sampling format	Adsorbent type	Adsorbent amount	Sample volume	Sampling Time	Washing	Elution	Targets	Detection system	LOD	Ref.
Sample preparation	Liquid samples	SPE / 1 mL-cartridge	Irregular inorganic polymer (25–36 nm)	50 mg	15 mL water sample	-	750 µL of water/MeOH mixture (40/60, v/v) and then drying for 5 min under vaccume	2 mL MeOH	2,4-DNT; TNT; 2,6-DNT; Tetryl	LC/UV	-	[31]
		SPE / 1 mL-cartridge	Irregular inorganic polymer (25–36 nm)	50 mg	15 mL water sample	-	water/ACN mixture (75/25, v/v) (1 mL)	1.5 mL Acetonitrile	2,4-DNT; TNT; 2,6-DNT; Tetryl	LC/UV	-	[37]
		Batch SPE-DLLME	Irregular inorganic polymer (50–80 nm)	100 mg	100 mL Wastewater sample	Batch SPE: 1 h	After removing the MIP, they washed with 2 mL water	2 mL MeOH/HOAC 7% and then DLLME was applied to this extract	3-NT	GC-FID	0.02 µg/L	[32]
		Batch SPE	Inhomogeneous spherical particles	1 mg/mL MIP in water sample contained 10% MeOH		30 min	-	MeOH 3 × 1mL	TNT; 2,4-DNT	IMS	TNT: 0.1 ppm 2,4-DNT:0.05 ppm	[33]
		SPE / 1 mL-cartridge	Hollow MIP spheres	100 mg	1 mL MeOH	10 min	1 mL Water/methanol (40/60, v/v) (30 s)	3 mL Acetonitrile; 1 mL methanol/acetic acid (80/20, v/v) (8 min)	HMX; RDX; TNT; CL-20	LC/UV	HMX:0.16, 0.28 RDX: 0.33, 0.36 TNT: 0.13, 0.19 CL-20:0.19, 0.32 µmol/L	[34]
		SPE/ flash chromatography (12 mL cartridge	Irregular aggregated particles	5 g	MeOH (up to 6 mL)	0.15 mL/min	MeOH (Up to 29 mL) 1 mL/min	Acetonitrile (Up to 13 mL) 1 mL/min	HMX; RDX	HPLC/DAD	HMX: 0.015 RDX:0.048 µmol/L	[35]
		SPE / 1 mL-cartridge	Irregular microparticles	100 mg	Acetonitrile (1 mL)	0.06 mL/min	MeOH (5 mL) 0.5 mL/min	Acetonitrile (3 mL) and methanol/acetic acid (4/1, v/v) (2 mL) 0.5 mL/min	CL-20	HPLC/DAD	-	[36]
	Gas samples	SPME	Uniform distributed MIP (thickness ≈ 50µm) along the entire silica support	-	From headspace of spiked rubble	40 min	-	Desorption at 250 °C and for 2 min	TNT	GC-MS	60 ng/Kg	[38]

Reference

1. Xie, C.G.; Zhang, Z.P.; Wang, D.P.; Guan, G.J.; Gao, D.M.; Liu, J.H. Surface molecular self-assembly strategy for tnt imprinting of polymer nanowire/nanotube arrays. *Anal. Chem.* **2006**, *78*, 8339–8346.
2. Xie, C.G.; Liu, B.H.; Wang, Z.Y.; Gao, D.M.; Guan, G.J.; Zhang, Z.P. Molecular imprinting at walls of silica nanotubes for tnt recognition. *Anal. Chem.* **2008**, *80*, 437–443.
3. Bunte, G.; Hurttlen, J.; Pontius, H.; Hartlieb, K.; Krause, H. Gas phase detection of explosives such as 2,4,6-trinitrotoluene by molecularly imprinted polymers. *Anal. Chim. Acta* **2007**, *591*, 49–56.
4. Walker, N.R.; Linman, M.J.; Timmers, M.M.; Dean, S.L.; Burkett, C.M.; Lloyd, J.A.; Keelor, J.D.; Baughman, B.M.; Edmiston, P.L. Selective detection of gas-phase tnt by integrated optical waveguide spectrometry using molecularly imprinted sol-gel sensing films. *Anal. Chim. Acta* **2007**, *593*, 82–91.
5. Edmiston, P.L.; Campbell, D.P.; Gottfried, D.S.; Baughman, J.; Timmers, M.M. Detection of vapor phase trinitrotoluene in the parts-per-trillion range using waveguide interferometry. *Sens. Actuators, B* **2010**, *143*, 574–582.
6. Riskin, M.; Tel-Vered, R.; Bourenko, T.; Granot, E.; Willner, I. Imprinting of molecular recognition sites through electropolymerization of functionalized au nanoparticles: Development of an electrochemical tnt sensor based on pi-donor-acceptor interactions. *J. Am. Chem. Soc.* **2008**, *130*, 9726–9733.
7. Riskin, M.; Tel-Vered, R.; Lioubashevski, O.; Willner, I. Ultrasensitive surface plasmon resonance detection of trinitrotoluene by a bis-aniline-cross-linked au nanoparticles composite. *J. Am. Chem. Soc.* **2009**, *131*, 7368–7378.
8. Riskin, M.; Tel-Vered, R.; Willner, I. Imprinted au-nanoparticle composites for the ultrasensitive surface plasmon resonance detection of hexahydro-1,3,5-trinitro-1,3,5-triazine (rdx). *Adv. Mater.* **2010**, *22*, 1387–+.
9. Riskin, M.; Ben-Amram, Y.; Tel-Vered, R.; Chegel, V.; Almog, J.; Willner, I. Molecularly imprinted au nanoparticles composites on au surfaces for the surface plasmon resonance detection of pentaerythritol tetranitrate, nitroglycerin, and ethylene glycol dinitrate. *Adv. Mater.* **2011**, *83*, 3082–3088.
10. Stringer, R.C.; Gangopadhyay, S.; Grant, S.A. Detection of nitroaromatic explosives using a fluorescent-labeled imprinted polymer. *Anal. Chem.* **2010**, *82*, 4015–4019.
11. Alizadeh, T.; Zare, M.; Ganjali, M.R.; Norouzi, P.; Tavana, B. A new molecularly imprinted polymer (mip)-based electrochemical sensor for monitoring 2,4,6-trinitrotoluene (tnt) in natural waters and soil samples. *Biosens. Bioelectron.* **2010**, *25*, 1166–1172.
12. Cennamo, N.; Dona, A.; Pallavicini, P.; D'Agostino, G.; Dacarro, G.; Zeni, L.; Pesavento, M. Sensitive detection of 2,4,6-trinitrotoluene by tridimensional monitoring of molecularly imprinted polymer with optical fiber and five-branched gold nanostars. *Sens. Actuators, B* **2015**, *208*, 291–298.
13. Cennamo, N.; D'Agostino, G.; Galatus, R.; Bibbo, L.; Pesavento, M.; Zeni, L. Sensors based on surface plasmon resonance in a plastic optical fiber for the detection of trinitrotoluene. *Sens. Actuators, B* **2013**, *188*, 221–226.
14. Yang, L.B.; Ma, L.A.; Chen, G.Y.; Liu, J.H.; Tian, Z.Q. Ultrasensitive sers detection of tnt by imprinting molecular recognition using a new type of stable substrate. *Chem.Eur. J.* **2010**, *16*, 12683–12693.
15. Holthoff, E.L.; Stratis-Cullum, D.N.; Hankus, M.E. A nanosensor for tnt detection based on molecularly imprinted polymers and surface enhanced raman scattering. *Sensors* **2011**, *11*, 2700–2714.
16. Nie, D.X.; Han, Z.; Yu, Y.Y.; Shi, G.Y. Composites of multiwalled carbon nanotubes/polyethyleneimine (mwcnts/pei) and molecularly imprinted polymers for dinitrotoluene recognition. *Sens. Actuators, B* **2016**, *224*, 584–591.

17. Alizadeh, T. Preparation of magnetic tnt-imprinted polymer nanoparticles and their accumulation onto magnetic carbon paste electrode for tnt determination. *Biosens. Bioelectron.* **2014**, *61*, 532–540.
18. Pesavento, M.; D'Agostino, G.; Alberti, G.; Biesuz, R.; Merli, D. Voltammetric platform for detection of 2,4,6-trinitrotoluene based on a molecularly imprinted polymer. *Anal. Bioanal. Chem.* **2013**, *405*, 3559–3570.
19. Shahdost-fard, F.; Roushani, M. Impedimetric detection of trinitrotoluene by using a glassy carbon electrode modified with a gold nanoparticle@fullerene composite and an aptamer-imprinted polydopamine. *Microchim. Acta* **2017**, *184*, 3997–4006.
20. Mamo, S.K.; Gonzalez-Rodriguez, J. Development of a molecularly imprinted polymer-based sensor for the electrochemical determination of triacetone triperoxide (tatp). *Sensors* **2014**, *14*, 23269–23282.
21. Huynh, T.P.; Sosnowska, M.; Sobczak, J.W.; Kc, C.B.; Nesterov, V.N.; D'Souza, F.; Kutner, W. Simultaneous chronoamperometry and piezoelectric microgravimetry determination of nitroaromatic explosives using molecularly imprinted thiophene polymers. *Anal. Chem.* **2013**, *85*, 8361–8368.
22. Zhu, W.; Tao, S.Y.; Tao, C.A.; Li, W.N.; Lin, C.X.; Li, M.; Wen, Y.Q.; Li, G.T. Hierarchically imprinted porous films for rapid and selective detection of explosives. *Langmuir* **2011**, *27*, 8451–8457.
23. Xu, S.F.; Lu, H.Z.; Li, J.H.; Song, X.L.; Wang, A.X.; Chen, L.X.; Han, S.B. Dummy molecularly imprinted polymers-capped cdte quantum dots for the fluorescent sensing of 2,4,6-trinitrotoluene. *Acs Appl. Mater. Inter.* **2013**, *5*, 8146–8154.
24. Xu, S.F.; Lu, H.Z. Ratiometric fluorescence and mesoporous structure dual signal amplification for sensitive and selective detection of tnt based on mip@qd fluorescence sensors. *Chem. Commun.* **2015**, *51*, 3200–3203.
25. Huynh, T.P.; Wojnarowicz, A.; Kelm, A.; Woznicki, P.; Borowicz, P.; Majka, A.; D'Souza, F.; Kutner, W. Chemosensor for selective determination of 2,4,6-trinitrophenol using a custom designed imprinted polymer recognition unit cross-linked to a fluorophore transducer. *Acs Sensors* **2016**, *1*, 636–639.
26. Dai, J.J.; Dong, X.Q.; de Cortalezzi, M.F. Molecularly imprinted polymers labeled with amino-functionalized carbon dots for fluorescent determination of 2,4-dinitrotoluene. *Microchim. Acta* **2017**, *184*, 1369–1377.
27. Xu, S.F.; Lu, H.Z. Mesoporous structured mips@cds fluorescence sensor for highly sensitive detection of tnt. *Biosens. Bioelectron.* **2016**, *85*, 950–956.
28. Apodaca, D.C.; Pernites, R.B.; Del Mundo, F.R.; Advincula, R.C. Detection of 2,4-dinitrotoluene (dnt) as a model system for nitroaromatic compounds via molecularly imprinted short-alkyl-chain sams. *Langmuir* **2011**, *27*, 6768–6779.
29. Guan, G.J.; Liu, R.Y.; Mei, Q.S.; Zhang, Z.P. Molecularly imprinted shells from polymer and xerogel matrices on polystyrene colloidal spheres. *Chemistry* **2012**, *18*, 4692–4698.
30. Hassanzadeh, J.; Khataee, A.; Oskoei, Y.M.; Fattahi, H.; Bagheri, N. Selective chemiluminescence method for the determination of trinitrotoluene based on molecularly imprinted polymer-capped zno quantum dots. *New J. Chem.* **2017**, *41*, 10659–10667.
31. Lordel, S.; Chapuis-Hugon, F.; Eudes, V.; Pichon, V. Development of imprinted materials for the selective extraction of nitroaromatic explosives. *J. Chromatogr. A* **2010**, *1217*, 6674–6680.
32. Ebrahimzadeh, H.; Abedi, H.; Yamini, Y.; Adlnasab, L. Molecular-imprinted polymer extraction combined with dispersive liquid-liquid micro-extractionfor ultra-preconcentration of mononitrotoluene. *J. Sep. Sci.* **2010**, *33*, 3759–3766.

33. Lu, W.; Li, H.Y.; Meng, Z.H.; Liang, X.X.; Xue, M.; Wang, Q.H.; Dong, X. Detection of nitrobenzene compounds in surface water by ion mobility spectrometry coupled with molecularly imprinted polymers. *J. Hazard. Mater.* **2014**, *280*, 588–594.
34. Wang, J.; Meng, Z.H.; Xue, M.; Qiu, L.L.; Dong, X.; Xu, Z.B.; He, X.; Liu, X.; Li, J.S. Simultaneous selective extraction of nitramine explosives using molecularly imprinted polymer hollow spheres from post blast samples. *New J. Chem.* **2017**, *41*, 1129–1136.
35. Wang, J.; Meng, Z.H.; Xue, M.; Qiu, L.L.; Zhang, C.F. Separation of 1,3,5,7-tetranitro-1,3,5,7-tetraazacyclooctane and 1,3,5-trinitro-1,3,5-triazacyclohexane by molecularly imprinted solid-phase extraction. *J. Sep. Sci.* **2017**, *40*, 1201–1208.
36. Wang, J.; Xue, M.; Meng, Z.H.; Xu, Z.B.; Luo, J. Application of molecularly imprinted polymers for the solid-phase extraction of hexanitrohexaazaisowurtzitane (cl-20) from soil samples. *Anal. Methods* **2016**, *8*, 4413–4420.
37. Lordel, S.; Chapuis-Hugon, F.; Eudes, V.; Pichon, V. Selective extraction of nitroaromatic explosives by using molecularly imprinted silica sorbents. *Anal. Bioanal.Chem.* **2011**, *399*, 449–458.
38. Bianchi, F.; Giannetto, M.; Mori, G.; D'Agostino, G.; Careri, M.; Mangia, A. Solid-phase microextraction of 2,4,6-trinitrotoluene using a molecularly imprinted-based fiber. *Anal. Bioanal.Chem.* **2012**, *403*, 2411–2418.
